# Structure of human GPR119-G_s_ complex binding APD597 and characterization of GPR119 binding agonists

**DOI:** 10.3389/fphar.2024.1310231

**Published:** 2024-01-15

**Authors:** Ruixue Li, Yuxia Qian, Jiening Wang, Zhen Han, Sheng Ye, Shan Wu, Anna Qiao

**Affiliations:** ^1^ School of Life Sciences, Tianjin University, Tianjin, China; ^2^ School of Life Sciences, Hubei University, Wuhan, Hubei, China

**Keywords:** cryo-electron microscope structure, GPR119, APD597, agonists, molecular docking

## Abstract

The rhodopsin-like receptor GPR119 plays a crucial role in glucose homeostasis and is an emerging target for the treatment of type 2 diabetes mellitus. In this study, we analyzed the structure of GPR119 with the agonist APD597 bound and in complex with the downstream G protein trimer by single particle cryo-electron microscopy (cryo-EM). Structural comparison in combination with function assay revealed the conservative and specific effects of different kinds of GPR119 agonists. The activation mechanism of GPR119 was analyzed by comparing the conformational changes between the inactive and active states. The interaction between APD597 derivatives and synthetic agonists with GPR119 was analyzed by molecular docking technique, and the necessary structural framework was obtained. The above conclusions can provide structural and theoretical basis for the development of therapeutic drugs for type 2 diabetes mellitus.

## 1 Introduction

The prevalence of diabetes, a chronic disease, has risen sharply in recent years, with type 2 diabetes emerging as the most prevalent form (Yang et al., 2017; Yu et al., 2019). It is estimated that the number of individuals with diabetes will reach 700 million by 2045 (Li et al., 2020). Diabetes not only has a profound impact on human health and quality of life, but also carries the potential for serious complications such as cardiovascular disease, kidney disease and psychiatric diseases, which can, in severe cases, lead to fatalities (Zhang et al., 2020). At present, diabetes management primarily relies on a range of hypoglycemic medications. However, many patients still struggle to achieve optimal blood sugar control, often encountering various adverse reactions. Therefore, there is a pressing need for the development of more scientifically rigorous and effective hypoglycemic drugs to meet the demands of patients.

Using bioinformatics techniques, the researchers determined that GPR119 belonged to the class A rhodopsin-like receptor (Fredriksson et al., 2003), which was more closely related to adenosine and cannabinoid receptors through phylogenetic analysis (Brown, 2007; Costanzi et al., 2008). The full-length human GPR119 protein consists of 335 amino acids, and homologous proteins have also been found in a variety of vertebrates, including rats, zebrafish, and monkeys (Overton et al., 2008). GPR119 is mainly expressed in gastrointestinal secretory cells and pancreatic beta cells (Chu et al., 2007), when GPR119 is activated, it will increase the accumulation of cAMP, increase the secretion of glucagon-like peptide-1 (GLP-1), glucose-dependent insulin peptide (GIP) or insulin (Dhayal and Morgan, 2010; Chepurny et al., 2016), which regulates the homeostasis of blood sugar levels in patients with type 2 diabetes. As a result, GPR119 it has become an emerging target for treatment of type 2 diabetes, and its agonist is expected to be a new type of hypoglycemic for treatment of type 2 diabetes (Li et al., 2021).

The efficacy of common endogenous agonists of GPR119 in cell experiments is relatively low, and its stability and solubility are poor, which is not conducive to clinical research (Ritter et al., 2016). The discovery of chemosynthetic agonists makes it possible to study the animal model of diabetes mellitus smoothly and to understand the mechanism of action more deeply. Among them, APD597 has good solubility compared with its structural analogue APD668, which produces high concentration of hydroxyl metabolites with long half-life after binding to the receptor (Semple et al., 2011), and can reduce the interaction between drugs, and can balance the agonist effect and intrinsic activity well (Semple et al., 2012). In order to better understand the binding characteristics of GPR119 and agonist and its activation mechanism, this paper analyzes the structure of GPR119 binding agonist APD597 and the downstream G-protein trimer complex interacting with it were analyzed by single particle cryo-electron microscopy technique, which can provide the structure and theoretical basis for the development of therapeutic drugs for type 2 diabetes therapeutic drugs.

## 2 Materials and methods

### 2.1 Expression and purification of APD597-GPR119-G_s_ complex

Human GPR119, Gα_s_, and Gβ1γ2 were co-expressed in High Five cells (Invitrogen) using an insect expression system. After a 48-hour expression period, cell precipitations were collected through centrifugation and then frozen for future use. The C terminus of GPR119 was tagged with strep, while the C terminus of Gβ1 has a his tag. Detailed construction information can be found in the previous article (Qian et al., 2022). Nb35 was constructed on pET-28a vector and expressed in *E. coli* (Invitrogen) (Rasmussen et al., 2011). The expressed bacteria were harvested, sonicated for cell lysis, and subsequently subjected to affinity chromatography and gel filtration chromatography to purify the target protein. The purified protein was concentrated and stored in liquid nitrogen for later use.

To prepare the collected cells for further processing, they were thawed and suspended in a solution containing 20 mM HEPES at pH 7.5, 50 mM NaCl, 2 mM MgCl_2_, 1‰ protease inhibitor (Aprotinin, and Leupeptin). Additionally, 10 μg/mL Nb35, 25 mU/mL apyrase, 10 µM APD597 were added. The suspension was ground and then incubated at 16°C for 1 h before being centrifuged at 38,000 rpm at 4°C for 30 min. After centrifugation, the supernatant was discarded, and the cell membrane was collected. The collected cell membranes were further processed by adding 20 mM HEPES at pH 7.5, 140 mM NaCl, 5 mM MgCl_2_, and 1‰ protease inhibitor (Aprotinin and Leupeptin). Additionally, 25 mU/mL Apyrase, 0.5% (w/v) lauryl maltose neopentyl glycol (LMNG), 0.025% cholesterol hemisuccinate (CHS), and 10 µM APD597 were added. The suspension was ground and then incubated at 4°C for 2 h before being centrifuged at 38,000 rpm at 4°C for 30 min. Following centrifugation, Strep-Tactin^®^ XT (IBA) resin was added, and the mixture was rotated overnight at 4°C. After the overnight incubation, the medium was centrifuged back into a purification column, and a solution containing 20 mM HEPES at pH 7.5, 140 mM NaCl, 5 mM MgCl_2_, 0.01% (w/v) LMNG, 0.0005% CHS, and 10 µM APD597 was used to remove impurities. The target protein complex was then eluted using a solution composed of 50 mM biotin, 100 mM Tris-HCl at pH 8.0, 150 mM NaCl, 5 mM MgCl_2_, 0.01% (w/v) LMNG, 0.0005% CHS, and 10 µM APD597. Following elution, the samples were concentrated and further purified by gel filtration chromatography using Superdex 200 Increase 10/300. The final samples were stored in a buffer containing 20 mM HEPES at pH 7.5, 100 mM NaCl, 0.01% (w/v) LMNG, 0.0005% CHS, 2 mM MgCl_2_, 10 µM APD597.

### 2.2 Cryo-EM grid preparation and data collection

To prepare the cryo-EM grids, we applied a 3 µL aliquot of the purified APD597-GPR119-G_s_ complex onto a glow-discharged 300 mesh holey carbon grids (Quantifoil R1.2/1.3, Au). These grids were then carefully blotted at 4°C under 100% humidity for 3 s, and subsequently plunge-frozen into liquid ethane using Vitrobot Mark IV (FEI).

Data acquisition was performed using a Titan Krios electron microscope operating at 300 kV and equipped with Gatan K3 direct electron detector and GIF Quantum energy filter. A total of 2.775 movies were collected at a nominal magnification of 105.000× in super-resolution mode with a binning of 2, resulting in a pixel size of 0.851Å. The defocus range used was −1.0 to −1.5 µm. The total electron dose of 54e^−^/Å^2^ was fractioned into 40 frames, with a cumulative exposure time of 2.5 s.

### 2.3 Data processing

All movies were imported and subjected to motion correction using MotionCor2 in Relion-3.0. Contrast Transfer Function (CTF) estimation was performed using Patch CTF Estimation in cryoSPARC. A total of 5,40,874 particles were picked by Blob Picker and was subsequently extracted with a binning of 2 for *ab initio* reconstruction and heterogeneous refinement. Templates generated by the dominate class was employed in Template Picker, resulting in the inclusion of 2,868,242 particles for further processing. After two rounds of *ab initio* reconstruction and heterogeneous refinement, the best class, consisting of 9,37,868 particles, was chosen and re-extracted for non-uniform refinement, which led to a final resolution of 2.80 Å.

### 2.4 Model building

The structure of APD597-GPR119-G_s_ complex was built using the structure of MBX-2982-GPR119-G_s_ Complex (PDB code: 7WCM) as an initial model. The model was rigidly fitted into cryo-EM density map using Chimera. Followed by iteratively manual building using COOT. Real-space refinement was conducted in PHENIX for model refinement and validation yielded the final model. Figures of density maps and model were prepared by Chimera.

### 2.5 Determination of GPR119 expression on cell surface

48 h after transfection, 10 μL cells were added with 15 μL anti-Flag M2-fluorescein isothiocyanate antibody (Sigma-Aldrich) at 4°C and incubated for 20 min away from light, and then 175 μL 1 × TBS was added to terminate the reaction. The expression of GPR119 on the cell surface was determined by flow cytometry FACSCalibur (Becton Dickinson, Sunnyvale, CA). The expression of negative cells without fluorescein isothiocyanate will be deducted from the final expression calculation. Data are from at least three independent experiments performed in triplicate.

### 2.6 cAMP accumulation assay

1 µg wild-type and mutant plasmid were transfected into human embryonic kidney 293 cells (Invitrogen), 48 h after transfection, 1 mL cells were cleaned with 1 × PBS and then treated with a stimulation buffer containing IBMX. After the cells were counted, 384-well plates were added with 5 μL per well, totaling 6,000 cells. Then added 5 μL of different concentration gradient APD597 and incubated at room temperature for 30min, and then added 5 μL each of cAMP Eu Cryptate reagent and cAMP d2 antibody were and incubated at room temperature and away from light for 1 h. Data was read using PerkinElmer. The dose-response curve was analyzed using GraphPad Prism 8.4.3 (GraphPad Software). Data are from at least three independent experiments performed in triplicate.

### 2.7 Molecular docking

In this experiment, AutoDock Vina software was used for docking. First, the preparation of the protein and ligand involved procedures such as hydrogenation, energy optimization. Ligand preparation included energy optimization, generation of protonated states, generation of 3D conformations, etc. The orthosteric site of the protein receptor was selected as the docking pocket, with a size of 10*10*10 Å^3^. Finally, the docking protocol was employed to dock the protein and ligand, and the suitable result was selected based on docking score and binding models.

## 3 Results

### 3.1 Structure of agonist APD597 and GPR119 complex

**FIGURE 1:**
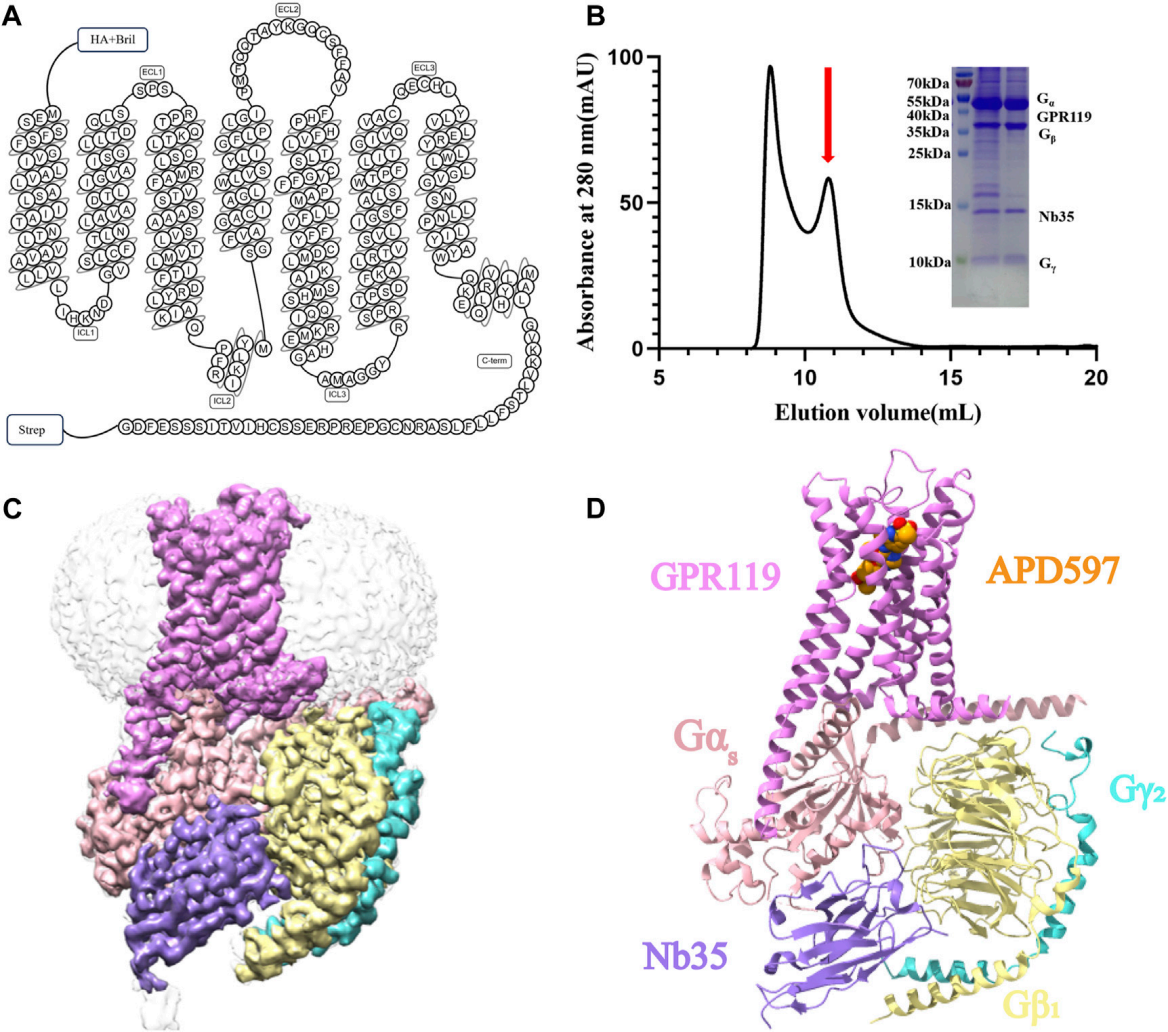
Overall structure of APD597-GPR119-Gs complex. **(A)** The sequence diagram of GPR119 was from GPCRdb. **(B)** The purification results of APD597-GPR119-G_s_ complex. **(C)** The cryo-EM density map of GPR119-G_s_-Nb35 in complex with APD597. **(D)** The cartoon representation of the complex structure shown in **(C)**.

GPR119 was modified to replace the native signal peptide with that of hemagglutinin (HA) and to add Bril at the N-terminus of GPR119 to ensure stable expression and proper folding, and the addition of a strep tag at the C-terminus for convenient purification (Figure 1A). Functional assays show that these modifications had little effect on ligand-binding and G_s_ activation of the receptor (Supplementary Figure S2). These modifications did not alter pharmacology of GPR119. To form an active, G protein-coupled complex, GPR119 was co-expressed with a dominant-negative Gα_s_ and Gβ1γ2 in HighFive insect cells. The complex was subsequently extracted and purified, with the addition of antibody Nb35 of Gα_s_ protein and the agonist APD597 to further stabilize the complex in its active state, using standard membrane protein purification protocols (Figure 1B). The APD597-GPR119-G_s_ structure was determined by cryo-EM single-particle analysis with an overall resolution of 2.8 Å. The high-quality cryo-EM map allowed us to model the 7TM elements of GPR119, the G_s_ heterotrimer, and Nb35 (Figure 1C). Importantly, APD597 was unambiguously identified in the orthosteric pocket of GPR119 (Figure 1D). Side chains of most residues are well defined except those of R213 to S219 of TM6. In addition, the density of the C-terminus (L301 to G335) of GPR119, and that of the α-helix domain of Gα_s_, were missing. GPR119 contains seven transmembrane helices and intracellular C-terminal domains connected by three extracellular regions and intracellular regions. The Gα_s_ protein lacks the density of the α-helix domain and forms an interaction interface with GPR119 through the Ras domain. GPR119 forms an interaction interface with Gβ1 through the first intracellular helix, and Nb35 stabilizes Gα_s_, Gβ1γ2. APD597 binds to a ligand binding pocket formed in the transmembrane helical region of GPR119 (Figure 1C, D). In order to further clarify the mechanism of agonist-receptor binding, the interaction characteristics of agonist APD597 with GPR119 are analyzed in detail below.

### 3.2 Interaction between agonist APD597 and GPR119

**FIGURE 2:**
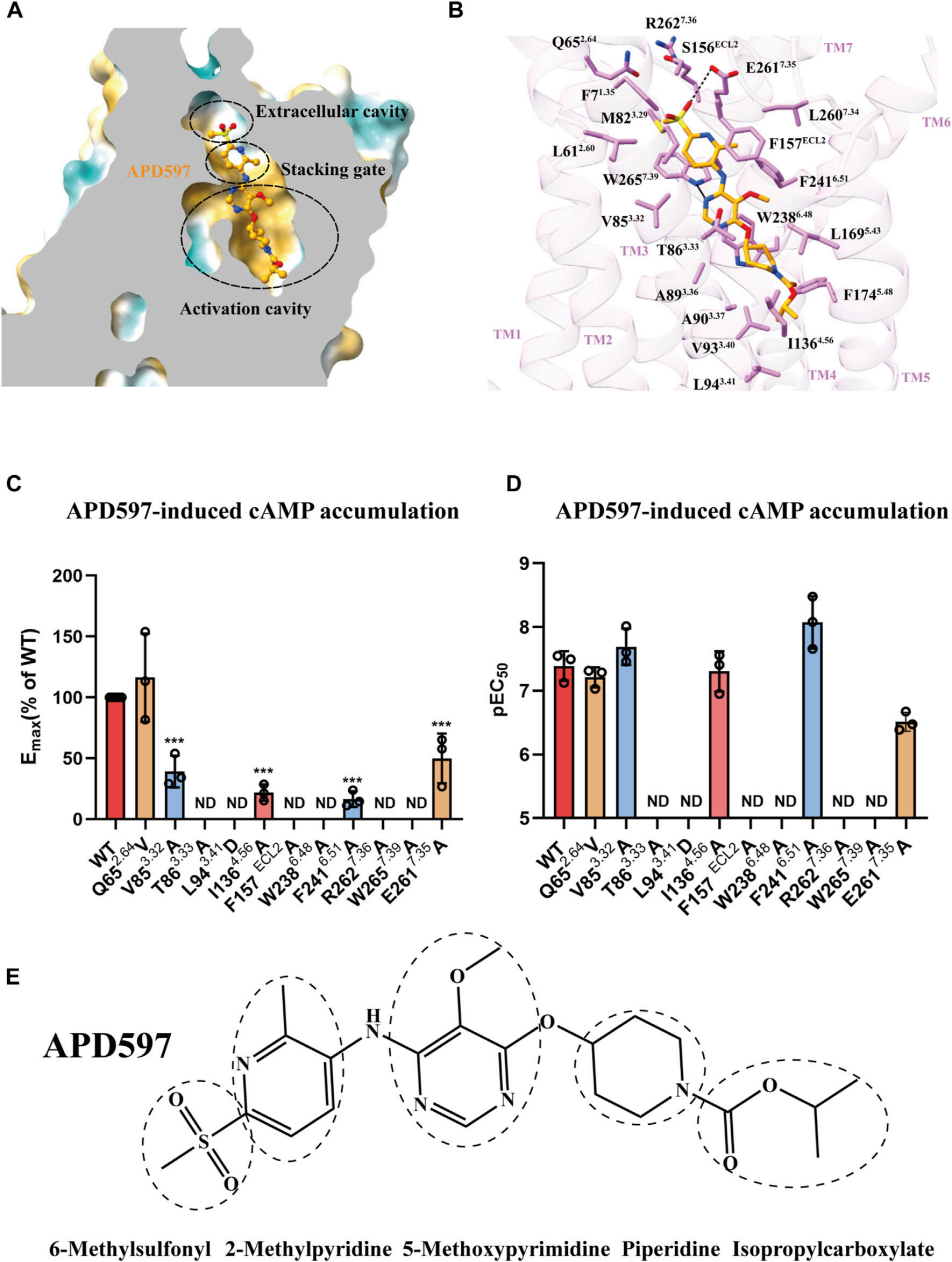
Interaction between agonist APD597 and GPR119. **(A)** The hydrophobic surface of agonist APD597 binding pocket. **(B)** The interaction between APD597 and the ligand binding pocket of GPR119. **(C–D)** The levels of cAMP accumulation in GPR119 mutants induced by APD597 relative to wild-type receptor. These data represent results obtained from a minimum of three independent experiments, each conducted in triplicate. Statistical analysis was performed using one-way ANOVA and Dunnett’s post-test, with significance denoted as ***p < 0.0001 when compared to the wild-type (WT) data. ND indicates cases where the response value was too low to detect. (wild type data shown in red, extracellular cavity mutants in orange, stacking gate mutants in blue, activation cavity mutants in pink). Further detailed data and expression information can be found in Supplementary Figure S2 and Supplementary Table S2. **(E)** Chemical structure formula of APD597.

**FIGURE 3:**
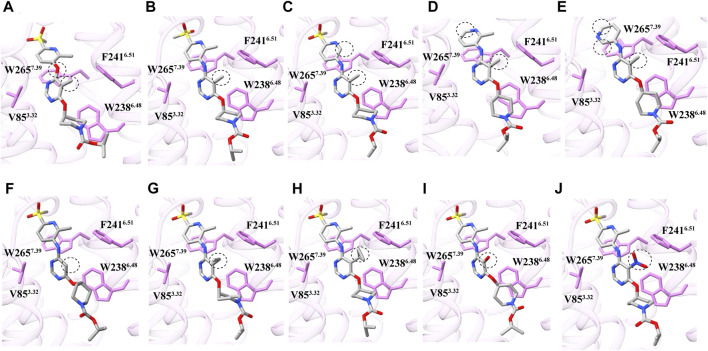
Docking model of different derivatives of APD597. **(A–J)** The dotted boxes are the different motifs of the derivatives, the EC_50_ are derived from other literature reports (Semple et al., 2012). The chemical structure of APD597 derivatives are shown in Supplementary Figure S3.

APD597 comprises three key components, the hexamethylsulfonyl group at the head, the intermediate dimethylpyridine and pentamethylpyrimidine, the tail piperidine and the carboxylic acid isopropyl ester. As shown in Figure 2A, the ligand binding pocket of GPR119 exhibits a pronounced hydrophobic nature. Based on its binding characteristics, this pocket can be divided into three regions, the extracellular cavity facing the outer membrane, a central stacking gate, and a hydrophobic activation cavity traversing the cell membrane, so named because it is proximity to the toggle switch residue W238^6.48^ (Qian et al., 2022). Figure 2B illustrates the interaction between APD597 and the GPR119 ligand binding pocket. APD597 binds to the extracellular cavity of the GPR119 ligand binding pocket through the hexamethylsulfonyl group, forming a hydrogen bond interaction with E261^7.35^, and is surrounded by Q65^2.64^, F7^1.35^, L61^2.60^, and E261^7.35^, R262^7.36^ and S156^ECL2^. To confirm the crucial roles, a series of mutant function experiments were conducted. Notably, when the polar amino acid R262^7.36^ was mutated to alanine, the agonist’s activation effect was completely abolished. Moreover, when E261^7.35^ was mutated to alanine, the half effective concentration (EC_50_) value decreased sixfold, accompanied by nearly a 50% reduction in cAMP accumulation. Interestingly, the agonist potency remained relatively unchanged when the polar amino acid Q65^2.64^ was mutated into a hydrophobic amino acid. These findings underscore the pivotal roles played by R262^7.36^ and E261^7.35^ (Figure 2C,D). Furthermore, it is worth noting that the hexamethylsulfonyl group exerts a significant influence on GPR119 activation. As indicated in Figure 3D,E, the removal of this group leads to a decrease in the EC_50_ value.

APD597 engages in π-π interactions with stacking gate residues F157^ECL2^ and W265^7.39^ through its dimethylpyridine moiety. Surrounding interactions involve residues M82^3.29^, V85^3.32^, F241^6.51^, and L260^7.34^. Upon individual mutations of F157^ECL2^ and W265^7.39^ to alanine, the activation response was completely abolished. Whereas the individual mutations of V85^3.32^ and F241^6.51^ to alanine resulted in a significant decrease in cAMP accumulation (Figure 2C,D). Notably, the methyl group with this compound plays a crucial role in GPR119 activation, as demonstrated in Figure 3C, E, the removal of this methyl group leads to a 20 to 100-fold reduction in its activation potency (Semple et al., 2012).

APD597 interacts with the hydrophobic activation cavity through the pentamethylpyrimidine tail, piperidine, and isopropyl carboxylate moieties, engaging a cluster of hydrophobic amino acids (T86^3.33^, A89^3.36^, A90^3.37^, V93^3.40^, L94^3.41^, I136^4.56^, and L169^5.43^). Notably, pentamethylcytosine forms a hydrogen bond with W265^7.39^, and engages in π-π interaction with W238^6.48^. The impact of the π-π interaction was inderscored when the mutation of W238^6.48^ to alanine resulted in a complete loss of activation effect. Furthermore, the nearly parallel orientation of T86^3.33^ to pentamethylcytosine and W238^6.48^ suggests a critical role. A mutation substituting the polar amino acid T86^3.33^ with non-polar glycine or alanine also abolished the agonist potency, potentially disrupting its interaction with pentamethylcytosine. Likewise, the substitution of the hydrophobic amino acid L94^3.41^ with negatively charged aspartic acid led to a complete loss of agonist potency, presumably by compromising the hydrophobic microenvironment essential for interaction. Additionally, mutating I136^4.56^ to alanine substantially reduced cAMP accumulation (Figures 2C,D). Notably, the nature of the 5-position of the central pyrimidinyl moiety substituents significantly influenced GPR119 activation, with this site displaying sensitivity to substituent size, particularly a limited tolerance for polar groups. As depicted in Figure 3F, smaller hydrogen atom substituents reduced the agonist potency by a factor of 50. Conversely, as the number of carbon atoms in the substituents increased to three or more, the activation effect gradually decreased (Figure 3A,B,G,H). Substituents with polar groups also resulted in a roughly 30-fold reduction in agonist potency (Figure 3I). Moreover, the 5-nitro substituent in the GPR119 prototype agonist AR231453, significantly diminished the agonist potency in the APD597 analog (Figure 3J).

### 3.3 Structural comparison of GPR119 complex with different agonists

**FIGURE 4:**
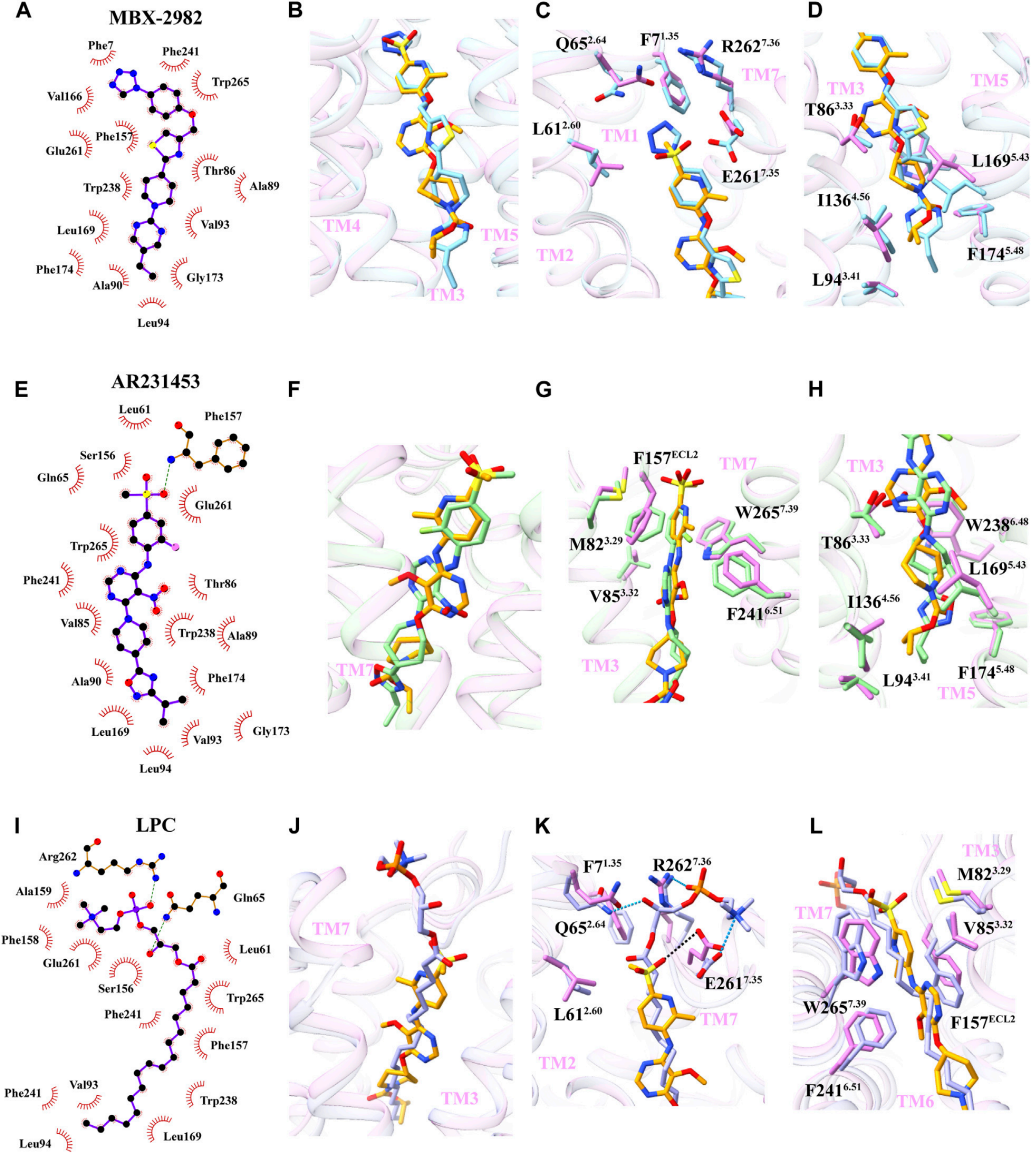
Structural comparison of APD597-GPR119 with MBX-2982-GPR119, AR231453-GPR119, LPC-GPR119. **(A, E and I)** The interaction between ligand (MBX-2982, AR231453, LPC) and GPR119 was analyzed by LigPlot^+^. **(B, F and J)** Overall structural comparison of APD597-GPR119 (violet) with MBX-2982-GPR119 (blue, PDB:7WCM), AR231453-GPR119 (green, PDB:7WCN), LPC-GPR119 (purple, PDB:7XZ5). **(C, D)** The conformational changes of residues between APD597-GPR119 with MBX-2982-GPR119. **(G, H)** The conformational changes of residues between APD597-GPR119 with AR231453-GPR119. **(K, L)** The conformational changes of residues between APD597-GPR119 with LPC-GPR119.

Structural comparison reveals that APD597, MBX-2982, and AR231453 adopt a similar binding mode in the orthosteric site of GPR119. This finding suggests a conserved mechanism of binding between agonists and GPR119. The GPR119 ligand binding pocket was divided into three distinct regions based on the binding characteristics of the agonist. A comparative analysis of the amino acids within these regions revealed that different agonists induce variations in the positioning of specific amino acids, resulting in varying agonist potency. In particular, when examining the structures of APD597 bound and MBX-2982 bound GPR119 complexes, it became evident that the amino acid conformation responsible for forming the central stacking gate remained largely unchanged (Figure 4B). However, the amino acid conformation associated with the extracellular cavity, namely Q65^2.64^ and E261^7.35^, exhibited noticeable deflections. Both of these amino acids came into closer proximity to the tetrazole moiety of MBX-2982 (Figure 4C). This observation was corroborated through mutation function experiments. Mutating Q65^2.64^ to valine significantly reduced the agonist potency, while mutating E261^7.35^ to alanine completely abolished the activation effect (Qian et al., 2022). In contrast, mutating Q65^2.64^ to valine had minimal impact on the agonist potency of APD597, while mutating E261^7.35^ to alanine reduced a portion of the agonist potency (Figures 2C,D). Additionally, I136^4.56^ and L169^5.43^ within the activation cavity displayed some bias, likely attributed to the larger ethylpyrimidine moiety of the hydrophobic interaction group in MBX-2982 compared to the isopropyl carboxylate moiety of APD597, resulting in stronger interaction effect (Figure 4D). Experimental results revealed that the agonist potency of MBX-2982 was entirely lost upon mutating I136^4.56^ to alanine (Qian et al., 2022), whereas the activation effect of APD597 was only reduced (Figures 2C,D). These findings shed light on the intricate structural and mechanistic aspects underlying the distinct agonist potency of these agonists, offering valuable insights into the development of therapeutic stragegies targeting type 2 diabetes mellitus.

Structural comparison of the APD597 bound and AR231453 bound GPR119 complex revealed notable conformational differences (Figure 4F). Specifically, the amino acids F157^ECL2^ and F241^6.51^, crucial components of the stacking gate, exhibited deviations (Figure 4G). In the case of AR231453, the 2-fluoro group engaged in a halogen-π interaction with F241^6.51^, while the 4-methylsulfonyl group forms a hydrogen bond with F157^ECL2^. Intriguingly, upon mutating F241^6.51^ to alanine, the agonist potency of AR231453 was entirely abolished, underscoring the strength of the interaction between AR231453 and GPR119 and the heightened agonist potency (Qian et al., 2022). Comparatively, the amino acids forming the extracellular and activation cavities displayed fewer deflections (Figure 4H).

Moreover, a comparison of the structures of APD597 bound and the endogenous agonist LPC bound GPR119 complexes revealed distinctive features. LPC, containing a more extensive head domain including cholinyl and phosphate groups in contrast to APD597, induced deflections in the amino acids of extracellular cavity, E261^7.35^, R262^7.36^, Q65^2.64^ and F7^1.35^. Additionally, the amino acid W265^7.39^, situated within the stack gate, exhibited deflection, possibly due to the π-π interaction involving the bound APD597 with F157^ECL2^ and W265^7.39^, facilitated by dimethylpyridine in the middle. This analysis underscores that various agonists bind to GPR119 through both conservative and specific mechanisms, resulting in distinct conformational changes and subsequent agonist potency (Figure 4I–L).

### 3.4 Mechanism analysis of GPR119 activation by APD597 and interaction between GPR119 and G_s_ protein

The activation of GPCR is involving the coupling of extracellular agonist binding and the recruitment of intracellular downstream effector proteins. This intricate process involves a conformational transition of the receptor from an inactive state to an activated state, facilitating the conversion of extracellular stimuli into cellular responses, a pivotal aspect for drug development. Given that GPR119 can be activated by endogenous agonists in the absence of exogenous ligand, elucidating the structure of the inactive receptor is challenging. In this study, we conducted a comparative analysis of the structure of the inactive state GPR119 predicted by AlphaFold (Uniprot ID: Q8TDV5) with the active state GPR119 structure in complex with APD597, to reveal a sequence of conformational alterations induced by GPR119 activation. To dissect the activation mechanism, we scrutinized the ligand-binding site residues and a set of conserved motifs integral to this activation process, namely PIF, NPxxY and DRY motifs. Our key findings are as follow: (1) For an agonist to bind, these hydrophobic contacts between F157^ECL2^ and W265^7.39^ referred as stacking gate and that between A89^3.36^ and the toggle switch residue W238^6.48^ which keep the receptor in an inactive state are broken, surrounding interactions residues M82^3.29^ and F241^6.51^ also display deflection (Supplementary Figure S4B). The ligand binding pocket undergoes an extensive structural rearrangement. (2) Within the PIF motif, the contact between W238^6.48^ and F234^6.44^ that keep the receptor in an inactive state is broken. Then the switching contacts of V93^3.40^ toward W238^6.48^ and A177^5.51^ toward F234^6.44^. In addition, the contacts between residues L230^6.40^ and I231^6.41^ with V96^3.43^ are broken and form contacts with residues F184^5.58^ and F181^5.55^, respectively. This reorganization loosens the contact of TM3-TM6 and facilitates the outward movement of the TM6. In addition, the conserved P176^5.50^ shift induces helical deformation, results in outward of TM5, which is significantly different to the inward bulge of TM5 observed in the canonical class A GPCRs (Supplementary Figure S4C). (3) The NPxxY and DRY motifs, Y279^7.53^ contacts with V25^1.53^ and V286^8.50^ are broken, and forms contacts with V96^3.43^, I99^3.46^ and R103^3.50^, moreover the residue R103^3.50^ display large deflection, this reorganization strengthens the contacts between TM3 and TM7 and loosens the contacts of TM3-TM6, drives the outward movement of TM6 (Supplementary Figure S4D). The cumulative effect of these amino acid shifts led to the displacement of the cytoplasmic end of TM5, TM6 outward, with the cytoplasmic end of TM7 moving inward. Consequently, a sufficiently spacious cavity was formed allowing the insertion of αH5 of Gα_s_ into the activated state receptor (Supplementary Figure S4A).

This comprehensive analysis sheds light on the intricate dynamics of GPR119 activation, providing crucial insights into the allosteric interplay between agonist binding and G protein coupling. Such insights are invaluable for advancing our understanding of GPCR activation and its implications for drug development targeting GPR119 in the context of type 2 diabetes mellitus.

**FIGURE 5:**
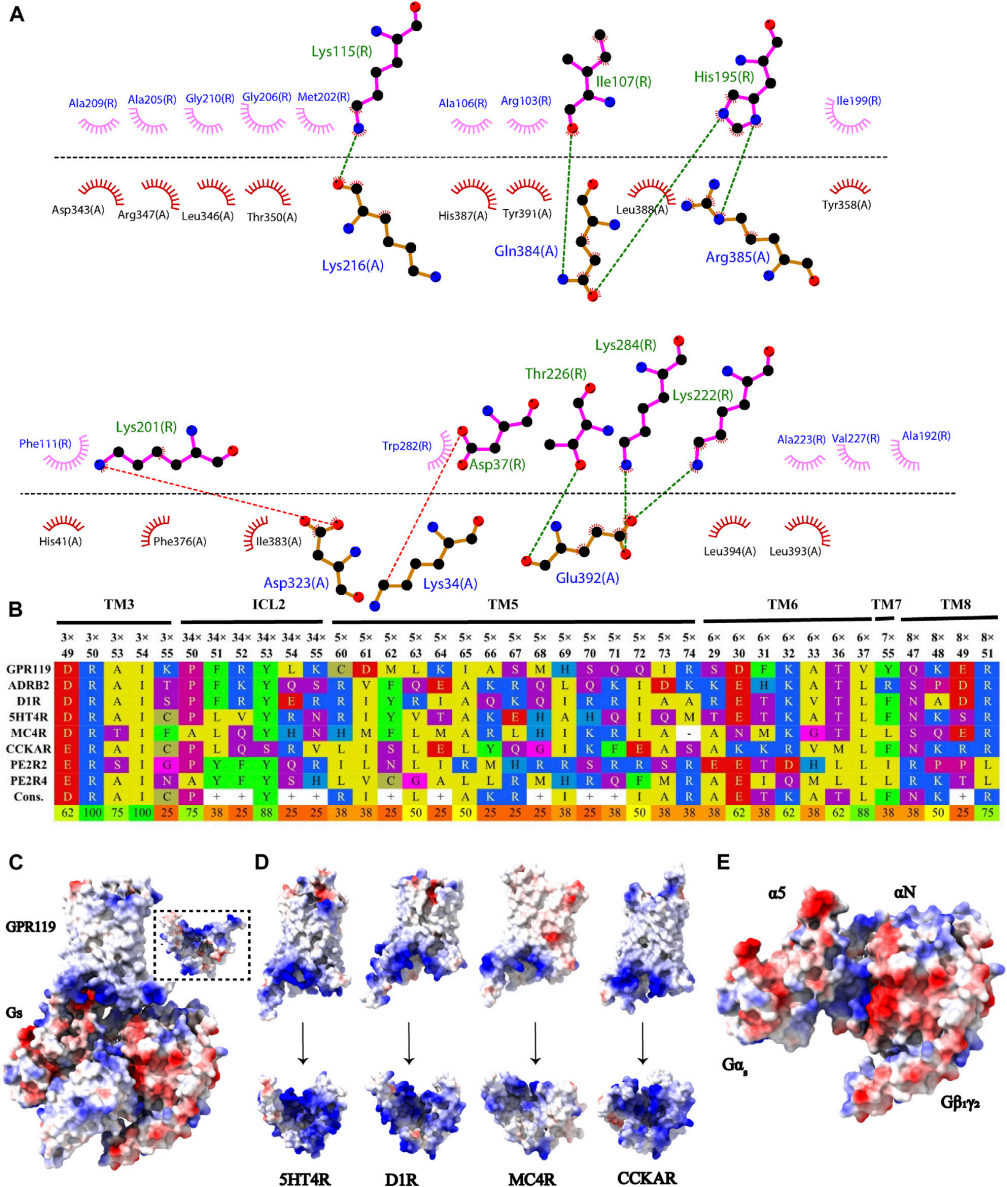
Interaction between GPR119 and G_s_ protein. **(A)** The interaction between GPR119 and G_s_ protein was analyzed by LigPlot^+^. GPR119 residues are located above the dashed black line, and G_s_ residues below the line. Hydrophobic interactions are illustrated by pink (GPR119) or red (G_s_) arcs. Amino acids involved in salt bridge and H-bonds are shown in atomic detail with salt bridge shown as dashed red lines and H-bonds shown as dashed green lines. **(B)** Sequence alignment of G_s_ protein interaction among different G_s_ -coupled class A GPCRs. The first line denote transmembrane helix numbering,34×denote ICL2, the second line denote Ballesteros–Weinstein numbering, the last line denote sequence identity. **(C)** Surface potential of GPR119-Gs complex, the dashed box is the GPR119 rotated 90° along the Y-axis. **(D)** Surface potential of different G_s_ protein-coupled class A GPCRs:5HT4R (PDB:7XT8), D1R (PDB:7JV5), MC4R (PDB:7PIU), CCKAR(PDB:7MBX), the arrow indicates that the corresponding receptor is rotated 90° along the Y-axis. **(E)** Surface potential of α5 and αN Helix of Gαs.

GPR1119 and Gα_s_ interact extensively through hydrogen bonds, salt bridges, and hydrophobic interactions. Where the α5 Helix of the Gα_s_ C-terminal inserts the pocket formed by GPR119 TM3, TM5 and TM6, the Q384^G.H5.16^, R385^G.H5.17^, E392^G.H5.24^ of Gα_s_ and the I107^3.54^, H195^5.69^, T226^6.36^, K284^8.48^, K222^6.32^ of GPR119 form hydrogen bond interactions. The H387^G.H5.19^, L388^G.H5.20^, Y391^G.H5.23^ of Gα_s_ and the R103^3.50^, A106^3.53^ of GPR119 form hydrophobic interactions, the F376^G.H5.08^, I383^G.H5.15^, L393^G.H5.25^, L394^G.H5.26^ of Gα_s_ and the F111^34.51^, W282^7.56^, A233^6.33^, V227^6.27^, A192^5.66^ of GPR119 form hydrophobic interactions. The D343^G.H4.13^, L346^G.H4.16^, R347^G.H4.17^, T350^G.h4s6.03^, Y358^G.h4s6.20^ of Gα_s_ Ras domain and the I199^5.73^, M202^5.76^, A205^5.79^, G206^5.80^, A209^ICL3^, G210^ICL3^ of GPR119 form hydrophobic interaction, D323^G.hgh4.13^ and K201^5.75^ form salt bridge. In addition, K34^G.HN.51^ of the Gα_s_ αN Helix forms a salt bridge with D37^ICL1^ of GPR119 (Figure 5A). By comparing different class A family receptor proteins with G_s_ interface (ADRB2:3SN6(Rasmussen et al., 2011), D1R: 7JV5 (Zhuang et al., 2021), 5HT4R: 7XT8 (Huang et al., 2022), MC4R: 7PIU(Heyder et al., 2021), CCKAR: 7MBX (Mobbs et al., 2021), PE2R2:7CX3 (Qu et al., 2021), PE2R4:7D7M (Nojima et al., 2021)) found that the formation of hydrophobic interactions of most highly conserved amino acids (Figure 5B), these conserved amino acids have a significant effect on the interaction between GPCR and G proteins, studies have shown that the mutation of the amino acid corresponding to F111^34.51^ to alanine can significantly reduce the interaction between GPCR and G proteins (Moro et al., 1993). After comparison, it was also found that only GPR119 and 5HT4R formed a salt bridge with Gβ_s_ through amino acids on ICL1, K35^ICL1^ of GPR119 and D323 of Gβ_s_, and R47^ICL1^ of 5HT4R and D312 of Gβ_s_ (Supplementary Figure S5), moreover, the study showed that the activation effect of GPR119 almost disappeared after the K35^ICL1^ mutation of GPR119 destroyed the interaction (Qian et al., 2022), indicating the specificity and importance of this interaction. In addition, through the analysis of the surface electrostatic potential of the complex protein, it was found that the electrostatic potential of the interface between GPR119 and G_s_ is complementary. The cytoplasmic part of TM3, ICL2, TM5, TM6, and TM7 of GPR119 are positive, while the α5 and αN Helix of Gα_s_ are negative. This potential complementation phenomenon is also highly conserved in the interactions of different class A family receptor proteins with G_s_ (5HT4R:7XT8, D1R:7JV5, MC4R:7PIU, CCKAR:7MBX). The complementary nature of amino acid sequence conservation and surface electrostatic potential conservation of class A family receptor proteins with G_s_ proteins reveals the conservative mechanism of GPCR receptor proteins' preference for binding to G_s_ proteins (Figure 5C–E).

### 3.5 Molecular docking of different synthetic agonists interacting with GPR119

**FIGURE 6:**
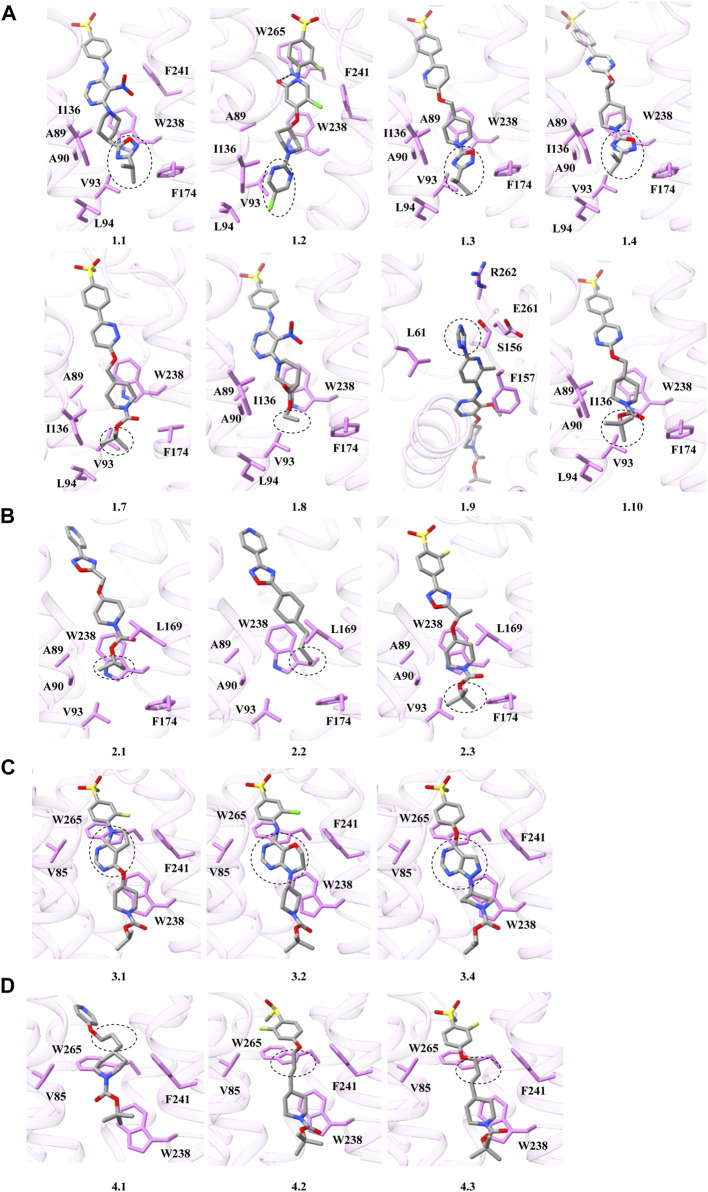
Docking model of different GPR119 synthesis agonists. **(A)** Six-membered heterocyclic core agonists. **(B)** Five-membered heterocyclic core agonists. **(C)** Double-ring fusion core agonists. **(D)** Linear connection core agonists. The dotted boxes are the different motifs of the synthesis agonists, the EC_50_ are derived from other literature reports (Jones et al., 2009; Shah and Kowalski, 2010; Buzard et al., 2012; Tyurenkov et al., 2018; Li et al., 2021; Qian et al., 2022). The chemical structure formula of GPR119 synthesis agonists are shown in Supplementary Figure S6; Supplementary Table S3.

The chemical structure of synthetic GPR119 agonists discovered thus far can generally be categorized into three components, a central core skeleton, a head motif containing aromatic rings, and a tail with lipophilic substituents. These core skeletons exhibit various types, including six-membered heterocyclic cores, five-membered heterocyclic cores, double-ring fusion cores and linear connection cores. The structural differences in these core types result in varying affinities with GPR119. Among the solved structures, agonists such as AR231453 and APD597 fall into the category of six-membered heterocyclic core agonists. Notable, agonists 1.1, 1.2, 1.3, 1.4, 1.10, and 1.7 demonstrate higher agonist potency on GPR119 compared to APD597. A close examination of their chemical structures reveals that these compounds all possess substantial lipophilic tails, enhancing their agonist potency. Conversely, 1.9 features a larger aromatic ring head motif, which facilitates binding with GPR119. However, 1.8 exhibits significantly reduced activation due to its smaller tail lipophilic substituents and the absence of substituents in the benzene ring in the head (Figure 6A). MBX-2982 represents a five-membered heterocyclic core agonist with a larger head containing a benzene ring and a lipophilic tail motif, which augments its binding to the ligand binding pocket of GPR119 compared to 2.1, 2.2, and 2.3, resulting in a higher activation effect (Figure 6B). Compared with 1.1, 1.3, and 1.4, their affinity is higher because the piperidine moiety in their tail connects oxadiazole moiety and forms a wider range of hydrophobic interactions (Figure 6A). APD668, on the other hand, is a bicyclic fusion core agonist. In comparison to 3.1, 3.2, and 3.4, their piperidine-linked pyrimidine motif is more favorable for forming π-π interaction with W238^6.48^ of GPR119, thus leading to higher activation (Figure 6C). As for linear-link core agonists 4.1, 4.2, and 4.3, their agonist potencies are notably reduced due to weakened interaction between the core region and GPR119 (Figure 6D). In summary, this analysis provides a comprehensive overview of the structural characteristics and activation properties of GPR119 agonists. This knowledge can guide the further refinement of existing agonists, enhancing their efficacy and activity, and serve as a foundational basis for the development of clinical drugs in the future.

## 4 Discussion

GPR119 has emerged as a promising target for type 2 diabetes, with its agonists holding potential as new hypoglycemic agent. Nevertheless, endogenous GPR119 agonists suffer from issues such as low potency, instability and poor solubility, which hinder their clinical applications. As a result, chemically synthesized agonists have been extensively developed. Among these, APD597 demonstrates favorable balance between agonist potency and intrinsic activity. It exhibits excellent solubility, reduced potency of drug-drug interactions, and without long-term sustained metabolite accumulation. However, its structural analog, APD668, binds to the receptor and generates hydroxyl metabolites with extended half-lives, posing potential risks associated with long-term accumulation. To address these concerns, we conducted a structural analysis of APD597-GPR119-G_s_ complex by single particle cryo-electron microscopy. By integrating the structural data with information on the structure-activity relationship (SAR) of APD597, we identified that specific substituents within different groups can significantly impact agonist activation, providing insight into further optimization and the development of novel compounds.

Comparative analysis of the structures of various agonists in complex with GPR119 revealed both commonalities and specific differences in their binding mechanisms. These differences contribute to variations in agonist potency, which hold promise for the generation of high-potency novel compounds. Additionally, we elucidated molecular mechanism within the agonist binding pocket and the G protein-coupled region by comparing the structures of the active and inactive GPR119 states. Currently, there are no GPR119-related drugs available on the market due to various challenges. Therefore, the comparative insights presented in this paper regarding various GPR119 binding compounds serve as valuable reference points, offering direction and information for drug development and optimization of GPR119-targeting therapies.

## Data Availability

The datasets presented in this study can be found in online repositories. The names of the repository/repositories and accession number(s) can be found in the article/Supplementary Material.

## Abbreviations

GPCR, G protein-coupled receptor; LMNG, lauryl maltose neopentyl glycol; CHS, cholesterol hemisuccinate; EC50, half effective concentration; SAR, structure activity relationship.
