# Supplementary material for: Structure of human GPR119-Gs complex binding APD597 and characterization of GPR119 binding agonists
Source: Front Pharmacol. 2024 Jan 15;15:1310231. doi: 10.3389/fphar.2024.1310231 (PMC10823026; doi:10.3389/fphar.2024.1310231)
Supplement: Supplementary file 1 [file Table1.docx]

Supplementary Material

Supplementary Figure 1 page S2

Supplementary Figure 2 page S4

Supplementary Figure 3 page S5

Supplementary Figure 4 page S5

Supplementary Figure 5 page S6

Supplementary Figure 6 page S7

Table S1 page S3

Table S2 page S4

Table S3 page S8

**
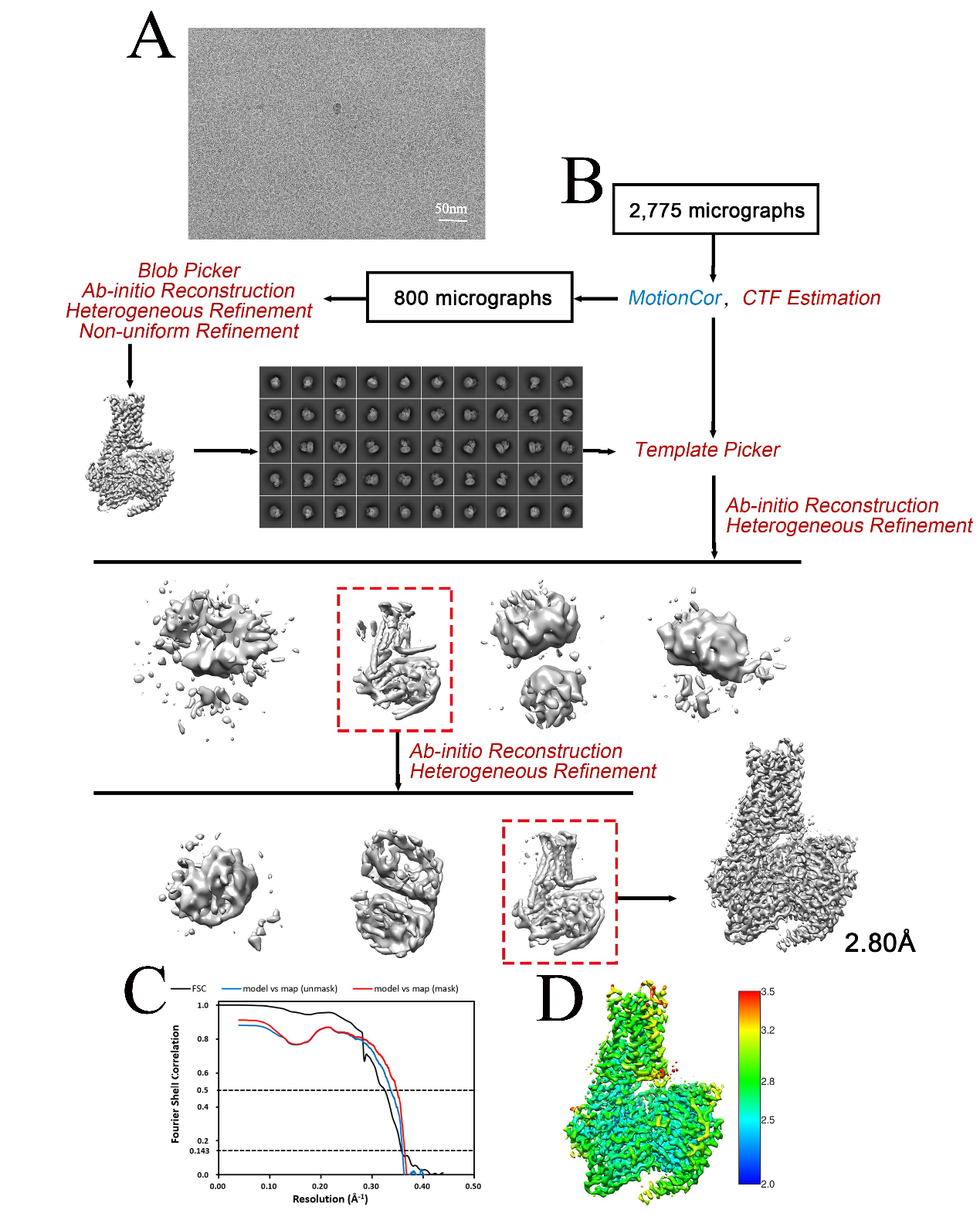
**

**Supplementary Figure 1. Cryo­-EM data processing of APD597-GPR119-G_s_ complex.**

**(A)** Representative 200kV cryo-electron microscope particle detection diagram, the scale is 50nm. **(B)** Cryo-electron microscopy image processing flow chart of APD597-GPR119-G_s_ complex. **(C)** Fourier shell correlation function curve of cryo-electron microscope. **(D)** Local resolution map of 3D classification model density map.

**Table S1.** **Structure statistics.**

| **Structure name** | **APD597-GPR119-G**_s_**-Nb35** |
| --- | --- |
| **Data collection and processing** |  |
| Magnification | 105,000 |
| Voltage (kV) | 300 |
| Electron exposure (e^-^/Å^2^) | 54 |
| Defocus range (μm) | -1.0 ~ -1.5 |
| Pixel size (Å) | 0.851 |
| Symmetry imposed | C1 |
| Initial particle projections (no.) | 2,868,242 |
| Final particle projections (no.) | 930,794 |
| Map resolution (Å) | 2.80 |
| FSC threshold | 0.143 |
| Map resolution range (Å) | 2.36 ~ 41.01 |
| **Refinement** |  |
| Initial model used | 7WCM |
| Model resolution (Å) | 2.87 |
| FSC threshold | 0.5 |
| Map sharpening B factor (Å^2^) | -130.8 |
| Model composition |  |
| Non-hydrogen atoms | 8,233 |
| Protein residues | 1,047 |
| Ligand | 1 |
| *B*-factors (Å^2^) |  |
| Protein | 1.91/97.74/33.87 |
| Ligand | 42.06/43.06/43.06 |
| R.m.s. deviations |  |
| Bond lengths (Å) | 0.003 |
| Bond angles (^o^) | 0.738 |
| Validation |  |
| MolProbity score | 1.35 |
| Clashscore | 3.24 |
| Rotamer outliers (%) | 0.00 |
| Ramachandran plot |  |
| Favored (%) | 96.41 |
| Allowed (%) | 3.59 |
| Disallowed (%) | 0.00 |


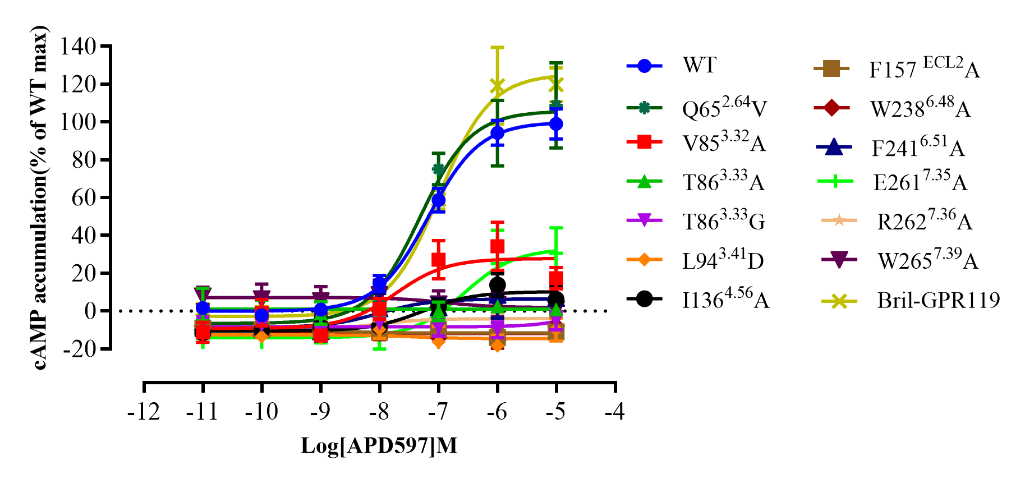


**Supplementary Figure 2. cAMP accumulation assay of GPR119.**

Amount of cAMP accumulation in GPR119 mutants induced by GPR119 relative to wild-type receptor, data are from at least three independent experiments performed in triplicate.

**Table S2. APD597 induced cAMP accumulation assays of GPR119.**

| **Mutants** | **APD597** | | | | | | **Expression** | |
| --- | --- | --- | --- | --- | --- | --- | --- | --- |
|  | **EC_50_**  **(nM)** | **_P_EC_50_** | | **Span** | | **n** |  |  |
|  |  | **mean ±s.e.m.** | ***P* value** | **% of WT** | ***P* value** |  | **% of WT** | ***P* value** |
| Wild type | 41.46 | 7.38±0.13 |  | 100±5 |  | 3 | 100 |  |
| Bril-GPR119 | 102.7 | 6.98±0.08 | 0.9955 | 120±4 | 0.073 | 3 | 124±15 | 0.9847 |
| Q65^2.64^V | 48.70 | 7.31±0.19 | 0.9998 | 116±10 | 0.2242 | 3 | 62±15 | 0.5560 |
| V85^3.32^A | 18.65 | 7.73±0.43 | 0.9827 | 38±7*** | ＜0.0001 | 3 | 55±8 | 0.2784 |
| T86^3.33^A | ND | ND | ND | ND | ND | 3 | 85±10 | >0.9999 |
| T86^3.33^G | ND | ND | ND | ND | ND | 3 | 40±11 | 0.0360 |
| L94^3.41^D | ND | ND | ND | ND | ND | 3 | 27±7* | 0.0045 |
| I136^4.56^A | 53.79 | 7.27±0.43 | 0.9997 | 21±4*** | ＜0.0001 | 3 | 65±11 | 0.6906 |
| F157 ^ECL2^A | ND | ND | ND | ND | ND | 3 | 73±18 | 0.9467 |
| W238^6.48^A | ND | ND | ND | ND | ND | 3 | 69±20 | 0.8468 |
| F241^6.51^A | 9.84 | 8.00±0.73 | 0.6926 | 15±5*** | ＜0.0001 | 3 | 61±20 | 0.5113 |
| E261^7.35^A | 272.1 | 6.56±0.44 | 0.3875 | 49±10*** | ＜0.0001 | 3 | 59±18 | 0.4256 |
| R262^7.36^A | ND | ND | ND | ND | ND | 3 | 102±7 | >0.9999 |
| W265^7.39^A | ND | ND | ND | ND | ND | 3 | 83±17 | 0.9997 |

Data are from at least three independent experiments performed in triplicate. ***P < 0.0001 performed by one-way ANOVA and Dunnett’s post-test, compared with the data of the WT. ND refers to the response value is too low to detect.


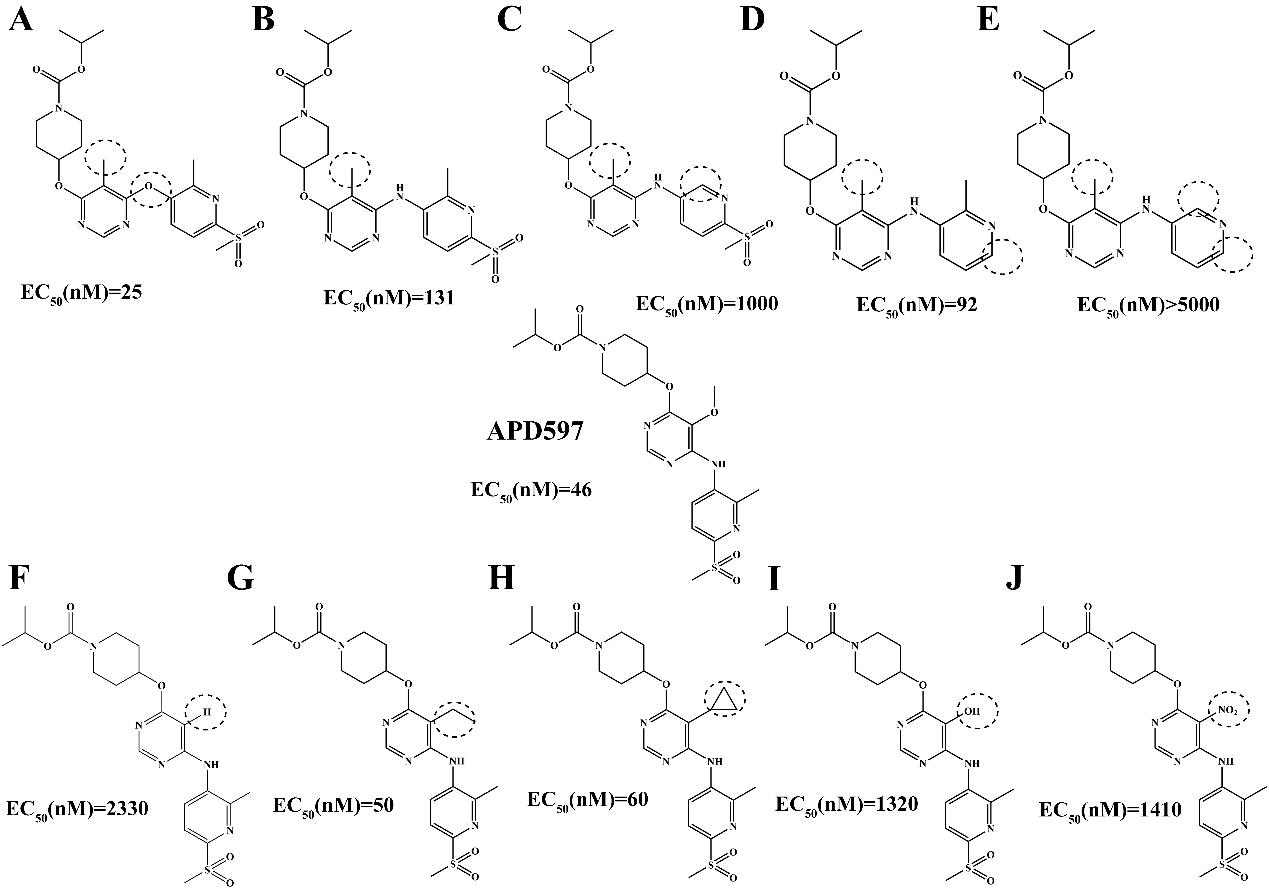


**Supplementary Figure3. Chemical structure of APD597 derivatives.**

**(A-J)** The dotted boxes are the different motifs of the derivatives, the EC_50_ are derived from other literature reports.


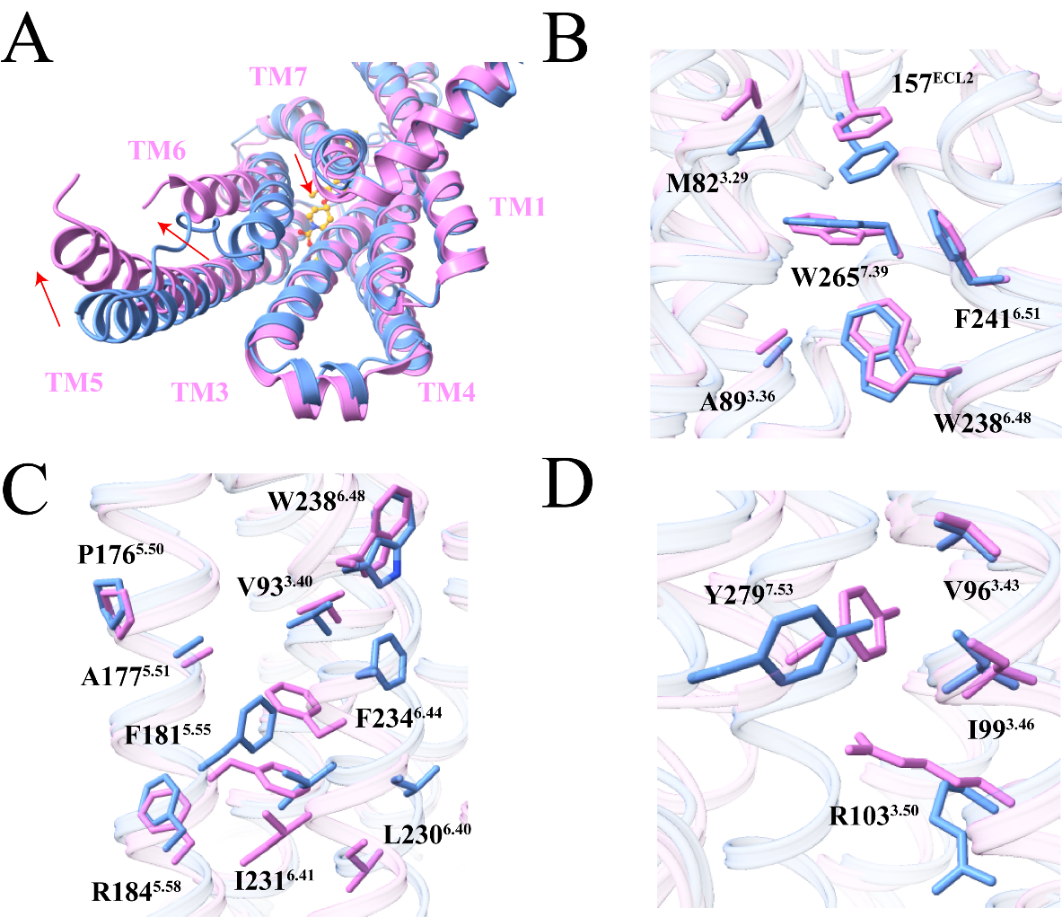


**Supplementary Figure4. Structural comparison of APD597-GPR119 and GPR119(predicted by AlphaFold).**

**(A)** Overall structural comparison of APD597-GPR119 and GPR119(predicted by AlphaFold), APD597-GPR119 in violet, GPR119(predicted by AlphaFold) in blue. **(B)** The conformational changes of ligand binding pocket residues between APD597-GPR119 with GPR119(predicted by AlphaFold). **(C-D)** The conformational changes of conserved motifs between APD597-GPR119 with GPR119(predicted by AlphaFold).


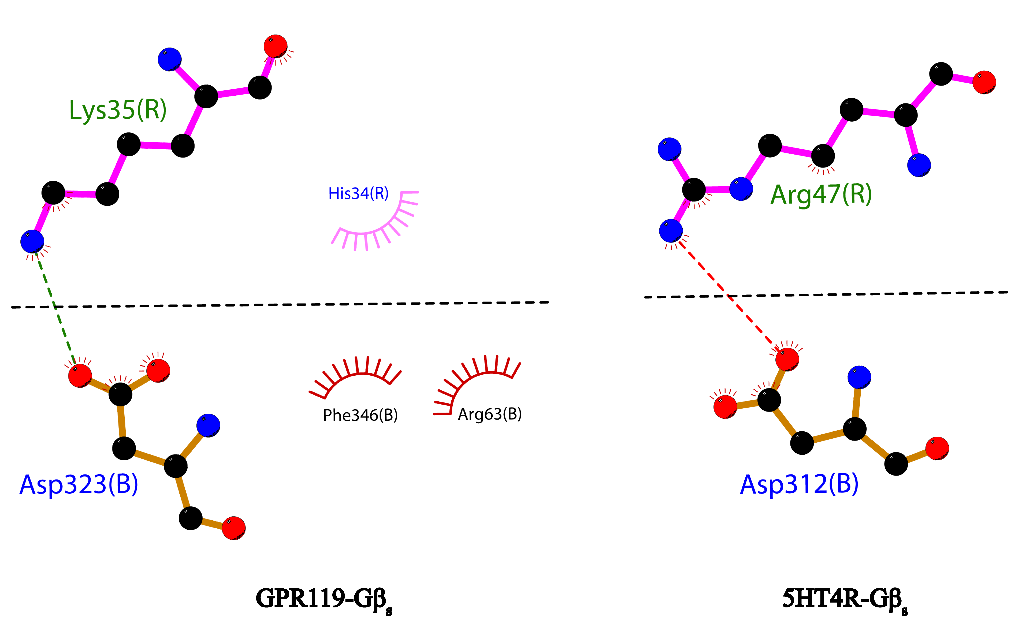


**Supplementary Figure5. Interaction between GPR119 and Gβ_s_ protein.**

The interaction between GPR119 and Gβ_s_ protein was analyzed by LigPlot^+^. GPR119 and 5HT4R residues are located above the dashed black line, and Gβ_s_ residues below the line. Hydrophobic interactions are illustrated by pink (GPR119) or red (Gβ_s_) arcs. Amino acids involved in salt bridge and H-bonds are shown in atomic detail with salt bridge shown as dashed red lines and H-bonds shown as dashed green lines.


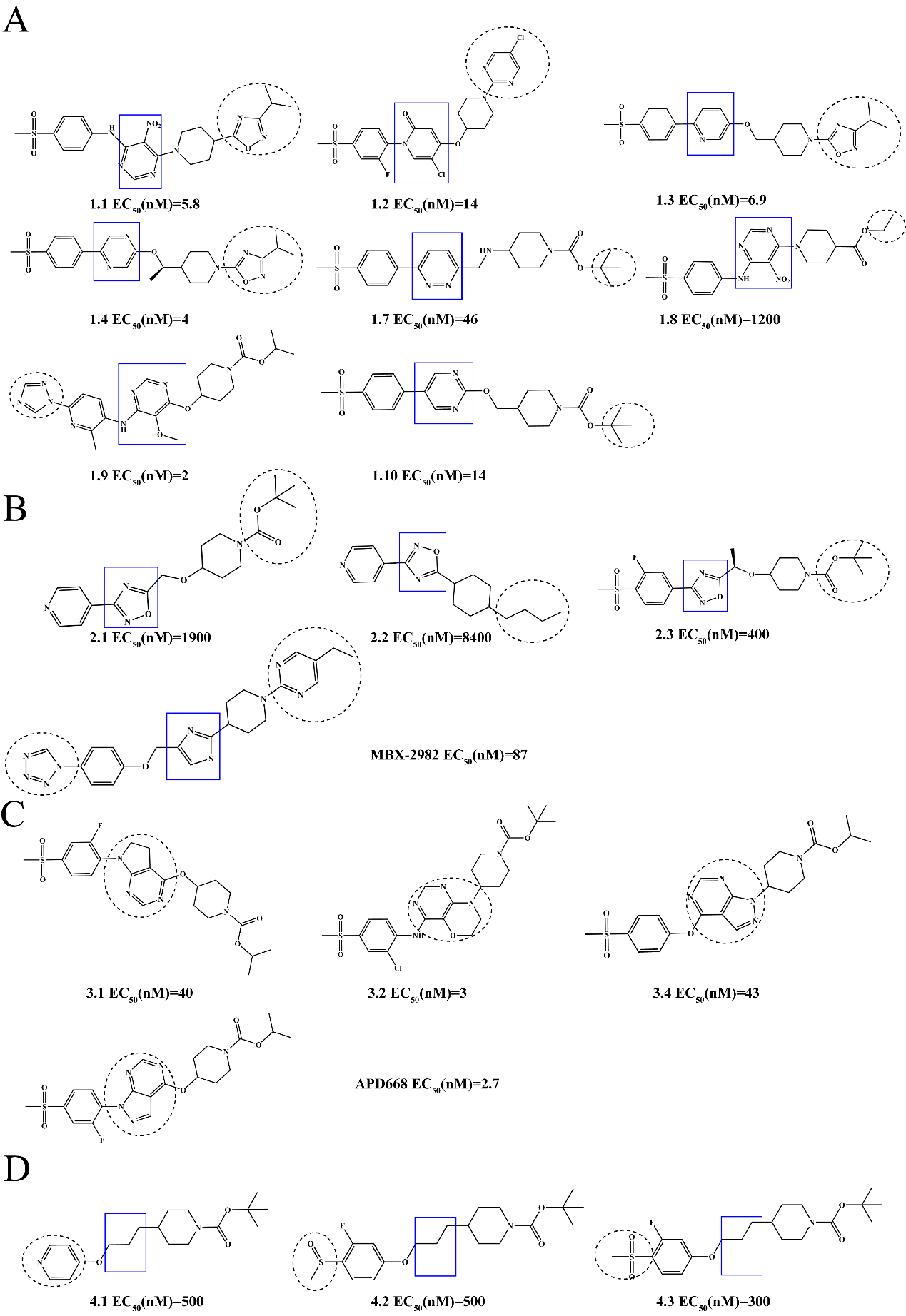


**Supplementary Figure6. Chemical structure formula of GPR119 synthesis agonists.**

**(A)** six-membered heterocyclic core agonists. **(B)** five-membered heterocyclic core agonists. **(C)** double-ring fusion core agonists. **(D)** linear connection core agonists. The blue boxes are the core skeleton, the black dotted circles are the different motifs of the synthesis agonists, the EC_50_ are derived from other literature reports(Jones et al., 2009; Shah and Kowalski, 2010; Buzard et al., 2012; Li et al., 2021; Qian et al., 2022).

**Table S3. Docking scores of synthetic agonists to GPR119.**

| **Ligand number** | **Docking Score** | **EC_50_** |
| --- | --- | --- |
| 1.1 | -11.69 | 5.8nM |
| 1.2 | -12.95 | 14nM |
| 1.3 | -12.02 | 6.9nM |
| 1.4 | -11.6 | 4nM |
| 1.7 | -11.24 | 46nM |
| 1.8 | -11.34 | 1.2µM |
| 1.9 | -10.84 | 2nM |
| 1.10 | -11.47 | 14nM |
| 2.1 | -10.03 | 1.9µM |
| 2.2 | -8.42 | 8.4µM |
| 2.3 | -11.85 | 0.4µM |
| 3.1 | -12.67 | 40nM |
| 3.2 | -13.14 | 3nM |
| 3.4 | -12.1 | 43nM |
| 4.1 | -8.51 | 0.5µM |
| 4.2 | -11.07 | 0.5µM |
| 4.3 | -10.63 | 0.3µM |
