# Supplementary figures and images for: Structure of human GPR119-Gs complex binding APD597 and characterization of GPR119 binding agonists
Source: Front Pharmacol. 2024 Jan 15;15:1310231. doi: 10.3389/fphar.2024.1310231 (PMC10823026; doi:10.3389/fphar.2024.1310231)

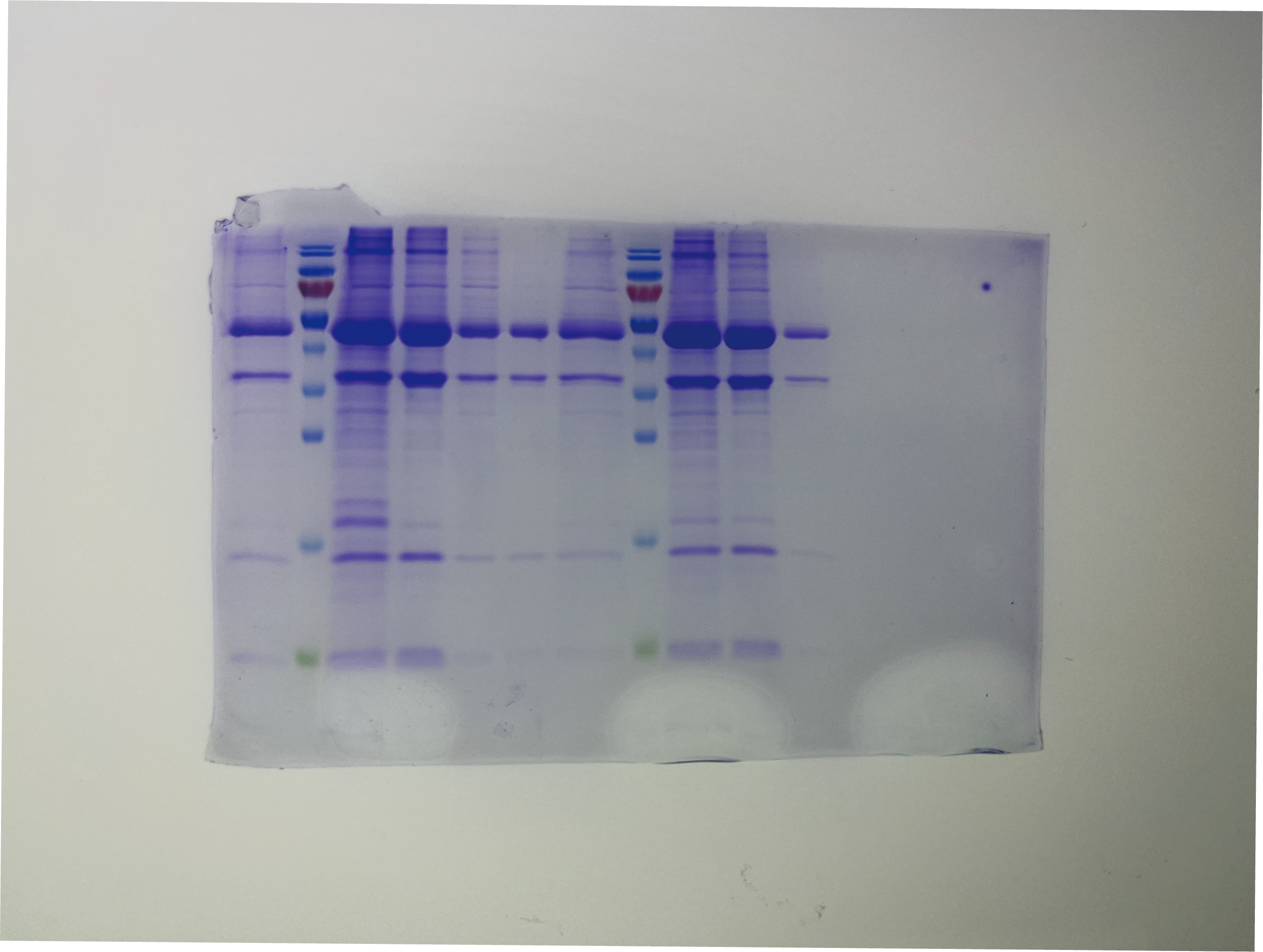

Supplement: Supplementary file 2 [file Image1.tif]
